# Targeting insect mitochondrial complex I for plant protection

**DOI:** 10.1111/pbi.12553

**Published:** 2016-03-17

**Authors:** Xiu‐Ming Wu, Chang‐Qing Yang, Ying‐Bo Mao, Ling‐Jian Wang, Xiao‐Xia Shangguan, Xiao‐Ya Chen

**Affiliations:** ^1^ National Key Laboratory of Plant Molecular Genetics and National Plant Gene Research Center (Shanghai)CAS Center for Excellence in Molecular Plant SciencesInstitute of Plant Physiology and EcologyShanghai Institutes for Biological SciencesChinese Academy of SciencesShanghaiChina; ^2^ Shanghai Key Laboratory of Plant Functional Genomics and ResourcesShanghai Chenshan Plant Science Research CenterChinese Academy of SciencesShanghai Chenshan Botanical GardenShanghaiChina

**Keywords:** *Helicoverpa armigera*, cotton, pest control, mitochondrial complex I, *NDUFV2*, RNA interference

## Abstract

Plant engineered to express double‐stranded RNA (dsRNA) can target the herbivorous insect gene for silencing. Although mounting evidence has emerged to support feasibility of this new pest control technology, field application is slow largely due to lack of potent targets. Here, we show that suppression of the gene encoding NDUFV2, a subunit of mitochondrial complex I that catalyses NADH dehydrogenation in respiratory chain, was highly lethal to insects. Feeding cotton bollworm (*Helicoverpa armigera*) larvae with transgenic cotton tissues expressing *NDUFV2* dsRNA led to mortality up to 80% within 5 days, and almost no larvae survived after 7 days of feeding, due to the altered mitochondrial structure and activity. Transcriptome comparisons showed a drastic repression of *dopa decarboxylase* genes. Reciprocal assays with Asian corn borer (*Ostrinia furnacalis*), another lepidopteran species, revealed the sequence‐specific effect of *NDUFV2* suppression. Furthermore, the hemipteran lugus *Apolygus lucorum* was also liable to *NDUFV2* repression. These data demonstrate that the mitochondrial complex I is a promising target with both sequence specificity and wide applicability for the development of new‐generation insect‐proof crops.

## Introduction

Insect pests cause enormous yield loss in crop production. Although chemical insecticides have brought great benefits to feed our growing population and support global economic sectors (Mascarelli, 2013; Stokstad, 2013; Verger and Boobis, 2013), there are increasing concerns on multiple environmental and human health problems (Heckel, 2012; Mascarelli, 2013; Stokstad, 2013; Ziska and McConnell, 2015). In the past 20 years, genetically engineered crops producing insecticidal proteins from *Bacillus thuringiensis* (Bt), mostly transgenic cotton, corn and canola, have contributed more and more to reducing insecticide usage and increasing yield and farmer profits (Carriere *et al*., 2015; Tabashnik *et al*., 2013); however, the limited scope of Bt susceptible insects and the rapid development of resistance call for new technologies of pest control (Carriere *et al*., 2015; Jin *et al*., 2015a; Tabashnik *et al*., 2013).

Plant‐mediated RNA interference (PM‐RNAi) engineers plant to express double‐stranded RNA (dsRNA) targeting to herbivorous the insect gene and suppress its expression after ingestion (Baum *et al*., 2007; Mao *et al*., 2007). It holds great promise for breeding the next‐generation insect‐proof crops due to the sequence‐specific preciseness and selectivity (Price and Gatehouse, 2008). Till now, this technology has been confirmed applicable to a wide range of chewing and piercing‐sucking insects, including cotton bollworm (*Helicoverpa armigera*), western corn rootworm (*Diabrotica virgifera virgifera* LeConte), Colorado potato beetle (*Leptinotarsa decemlineata*) and the English grain aphid (*Sitobion avenae*) (Abdellatef *et al*., 2015; Baum *et al*., 2007; Mao *et al*., 2007, 2011; Zhang *et al*., 2015). Our previous report showed that the RNAi effect could be enhanced by co‐expressing cysteine protease to increase the permeability of insect midgut peritrophic matrix (Mao *et al*., 2013). More recently, an important progress has been made in engineering plastids to express high dosage of the dsRNA (Jin *et al*., 2015b; Zhang *et al*., 2015). However, most genes examined so far show moderate inhibitive effects on insect growth and development, and highly potent targets are in urgent demand for either nucleic or plastid dsRNA expression.

Mitochondrial NADH:ubiquinone oxidoreductase (complex I) is one of the largest membrane‐bound protein assemblies with 14 central and up to 32 accessory subunits, containing flavine mononucleotide and iron–sulphur clusters as redox prosthetic groups (Brandt, 2006; Hirst, 2013). It pumps protons across the inner membrane of mitochondria and generates proton gradient to drive ATP production (Brandt, 2006), and is also an important source of reactive oxygen species (ROSs) (Sena and Chandel, 2012). Dysfunctions of human complex I subunits may lead to imbalance of NADH/NAD+ ratios and ROS production (Kussmaul and Hirst, 2006), causing Leigh's syndrome, mitochondrial encephalomyopathy, lactic acidosis and stroke‐like episodes (MELAS) (Benit *et al*., 2003; Koene *et al*., 2012).

Here, we report that suppression of *NDUFV2*, which encodes the 24‐kD subunit of mitochondrial complex I, was lethal to lepidopteran and hemipteran insects, and the effect was species specific. Thus, *NDUFV2* can serve as a promising target for further development of PM‐RNAi for precise insect pest control.

## Results and Discussion

### Identification of *NDUFV2* as a target gene for PM‐RNAi

We employed cotton bollworm (*H. armigera*), a lepidopteran generalist insect and one of the most devastating agriculture pests, to screen for effective PM‐RNAi target genes that can quickly control larval growth or induce high mortality. Based on our previous assembly and annotation of *H. armigera* EST data set (Tao *et al*., 2012), 50 genes involved in metabolism, energy production and hormone signalling were selected, and their corresponding dsRNAs were expressed in *Arabidopsis* plants under the control of CaMV 35S promoter. Rosette leaves from two to five homozygous transgenic lines with high dsRNA levels were used to feed the 2nd‐instar larvae.

**Figure 1:**
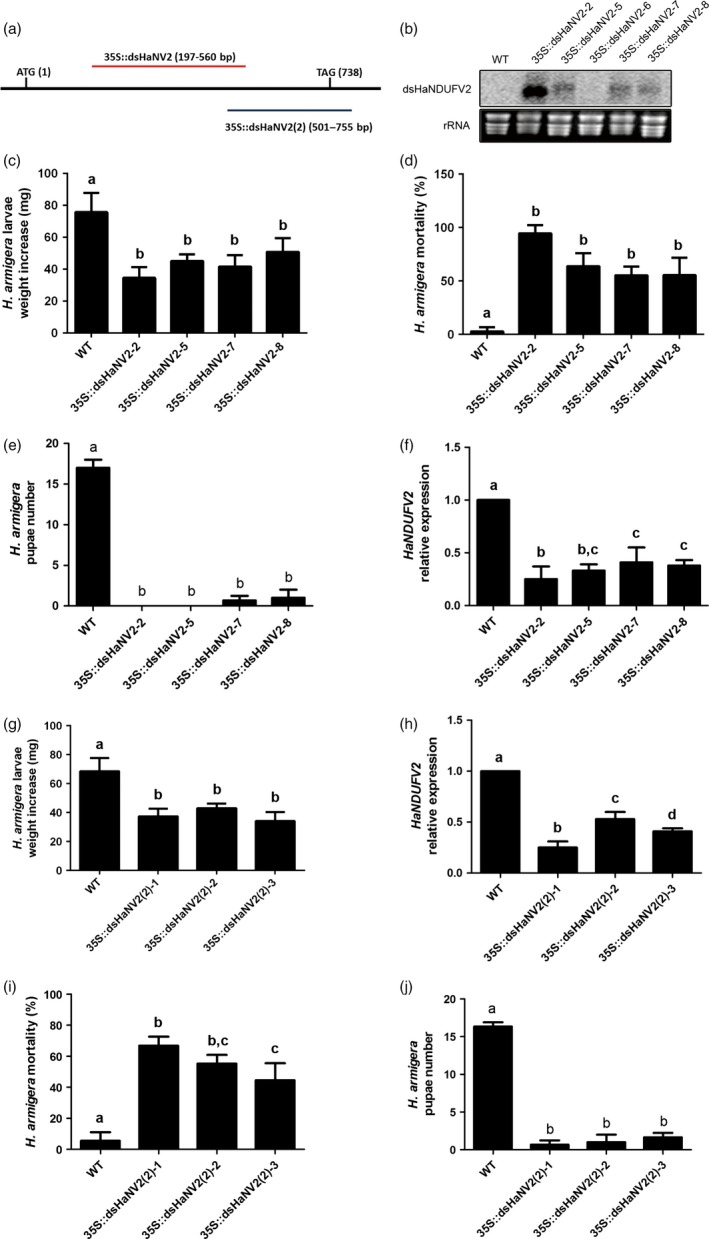
Suppression of *Helicoverpa armigera* larval growth by *35S::dsHaNV2* and *35S::dsHaNV2(2) Arabidopsis*. (a) Schematic show of fragments used in making the produce *35S::dsHaNV2* (red line) and *35S::dsHaNV2(2)* (blue line) constructs. (b) Transcript abundance of *dsHaNDUFV2* dsRNA in transgenic *Arabidopsis*. Ten micrograms of total RNAs from *35S::dsHaNV2* or wild‐type (WT) *Arabidopsis* leaves was separated on 1% agrose gel, transferred to Hybond N+ membrane and probed with ^32^P labelled *HaNDUFV2* fragment. (c) Average weight increase of larvae fed with leaves of different *35S::dsHaNV2 Arabidopsis* lines for 5 days. Each treatment started with 18 larvae. Data are mean ± SD of survivals of three replicates. (d) Mortality of larvae after feeding wild‐type (WT) or *35S::dsHaNV2 Arabidopsis* leaves for 14 days. Four transgenic lines were used to feed the 2nd‐instar larvae. Each treatment started with 18 larvae. Data are mean ± SD of three biological replicates. (e) Pupae number after feeding *H. armigera* with WT or *35S::dsHaNV2 Arabidopsis* leaves. Each treatment started with 18 larvae. Data are mean ± SD of three biological replicates. (f) Expression of *HaNDUFV2* in midgut of larvae fed with WT or *35S::dsHaNV2 Arabidopsis* leaves for 6 days. Data are mean ± SD of three biological replicate. (g) Weight increase of larvae after feeding *35S::dsHaNV2(2) Arabidopsis* leaves for 5 days. Each treatment started with 18 larvae. (h) Expression of *HaNDUFV2* in midgut of larvae fed with WT or *35S::dsHaNV2(2) Arabidopsis* leaves for 5 days. Data are mean ± SD of three biological replicates. (i) Mortality of larvae after feeding WT or *35S::dsHaNV2(2) Arabidopsis* leaves for 14 days. Three transgenic lines were used to feed the 2nd‐instar larvae. Each treatment started with 18 larvae. Data are mean ± SD of three biological replicates. (j) Pupae number after feeding *H. armigera* with WT or *35S::dsHaNV2(2) Arabidopsis* leaves. Each treatment started with 18 larvae. Data are mean ± SD of three biological replicates. Shared lowercase letters on panels (c–j) indicate no significant difference between the groups by one‐way ANOVA with Tukey's HSD test at confidence level of *P* < 0.05.

Of these, *HaNDUFV2*, encoding the subunit NDUFV2 of the mitochondria complex I (NADH:ubiquinone oxidoreductase), exhibited a high activity in inhibiting larval growth. The open reading frame (ORF) of HaNDUFV2 is 738 bp long, from which a 364‐bp fragment (197–560 bp) was expressed for dsRNA production in transgenic *Arabidopsis* plants (Figure 1a,b). After feeding with the *35S::dsHaNV2* leaves for 5 days, larvae exhibited severely retarded growth as revealed by 40–60% less weight compared with the control groups, which were administrated with leaves taken from nontransgenic (wild‐type, WT) or transgenic plants harbouring other dsRNA constructs (Figure 1c and Figure S1 a,b). The growth inhibition became observable within 2 days of the feeding, and progressively severer along with the assay time (Figure S1 b). After 14 days of feeding, mortalities ranged from 55 to 95% in tested groups, in comparison with 5–10% mortality of those fed with WT plants (Figure 1d). Pupation of larvae was observed after continuous feeding of WT plants for 25–30 days (Figure 1e, Table S1). For those fed with transgenic plants, however, most of the larvae died before pupation. When the limited survivals were able to pupate, no eclosion was observed (Figure 1e, Table S1). Quantitative RT‐PCR (qRT‐PCR) analysis showed that expression of *HaNDUFV2* in midgut was clearly suppressed by the *35S::dsHaNV2* leaves (Figure 1f).

To further examine the effect of plant‐mediated *HaNDUFV2* suppression, we selected another fragment of this gene, *HaNV2(2)* (501–755 bp), to generate dsRNA in *Arabidopsis* (Figure 1a). Similar inhibition on larval growth and *HaNDUFV2* gene expression was observed (Figure 1g,h), and feeding of *35S::dsHaNV2(2)* transgenic plants for 2 weeks also led to mortality up to 70% (Figure 1i). After completion of the feeding (~30 days), the pupae numbers of the test groups were greatly reduced compared with the control (Figure 1j). These data demonstrate that *HaNDUFV2* is a potent gene for effective PM‐RNAi against cotton bollworm.

### *35S:HaNDUFV2* cotton plants are resistant to bollworm larval damage

**Figure 2:**
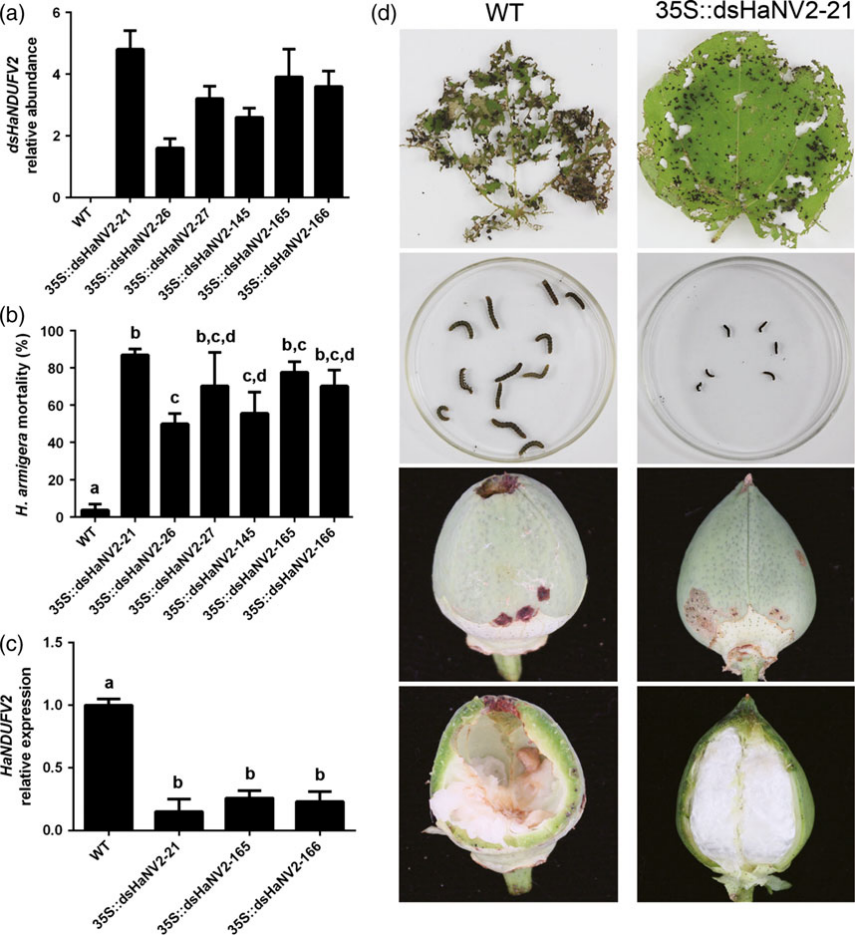
*35S::dsHaNV2* cotton plants were resistant to *Helicoverpa armigera* larvae. (a) Expression of *dsHaNDUFV2* in leaves of *35S::dsHaNV2* cotton lines. Data are mean ± SD of three biological replicates. (b) Mortality of larvae after feeding different *35S::dsHaNV2* cotton lines for 5 days. Leaves were used to feed 2nd‐instar larvae. Each treatment contained 18 larvae. Data are mean ± SD of three biological replicates. (c) Relative expression of *HaNDUFV2* in midgut of 3rd‐instar larvae survived after feeding WT or *35S::dsHaNV2* cotton leaves for 3 days. Data are mean ± SD of three biological replicates. (d) Enhanced resistance of *35S::dsHaNV2* cotton to *H. armigera*. Leaves of nontransgenic or *35S::dsHaNV2* cottons were used to feed 2nd‐instar larvae (12 larvae each treatment) for 3 days. Survivals after the assay are shown. Bolls of WT or *35S::dsHaNV2* cottons were used to feed 3rd‐instar larvae for 7 days, and the resultant bolls are shown. Each test repeated >3 times. Shared lowercase letters on panels (b) and (c) indicate no significant difference between the groups by one‐way ANOVA with Tukey's HSD test at confidence level of *P* < 0.05.

We next asked whether the PM‐RNAi towards *NDUFV2* was also or even more effective in the preferred host plant of the target insect. The *35S::dsHaNV2* construct was introduced into cotton (*G. hirsutum* cv. R15), and the transgenic lines with high dsRNA expressions were selected (Figure 2a, Figure S2). Strikingly, compared with *Arabidopsis*, the transgenic cotton plants showed a much stronger effect on larval growth and mortality, with only 5 days required to achieve lethality of 50–80% in test populations, and after 7 days, almost no larvae survived from the *35S::dsHaNV2* cotton leaves (Figure 2b, Figure S3 a). Survivals at day 5 exhibited little growth and were 4–5 times smaller than those fed with nontransgenic cotton leaves (Figure S3 b). Consistent with larval growth inhibition, the *HaNDUFV2* expression in midgut was markedly suppressed by the transgenic cotton leaf (Figure 2c). Furthermore, this insect resistance trait was stably transmitted to progenies, at least to the T_3_ generation we have obtained so far.

The protection of cotton leaves and cotton bolls (fruits) from bollworms was then examined. The 3rd‐instar larvae were placed on leaves, and after 3 days, almost all leaf tissues of the wild type were ingested, whereas larvae preceded less damage on the *35S::dsHaNV2* transgenic cotton leaves (Figure 2d). In contrast to the well growth of larvae raised on nontransgenic cotton leaves, those on *35S::dsHaNV2* showed extremely slow growth and 40–50% mortality (Figure 2d). When the 15‐DPA cotton bolls were given to the larvae, most larvae drilled into the wild‐type bolls from top and ate the majority of ovules after 7 days. However, less damage was observed on the *35S::dsHaNV2* cotton bolls, and most larvae ingested only limited epidermal tissues of the pericarp, causing little or no damage to ovules, and their growth was slow or ceased (Figure 2d). These results demonstrate that the *35S::dsHaNV2* cotton plants were highly resistant to cotton bollworm.

### PM‐RNAi of *NDUFV2* leads to mitochondria dysfunction midgut

**Figure 3:**
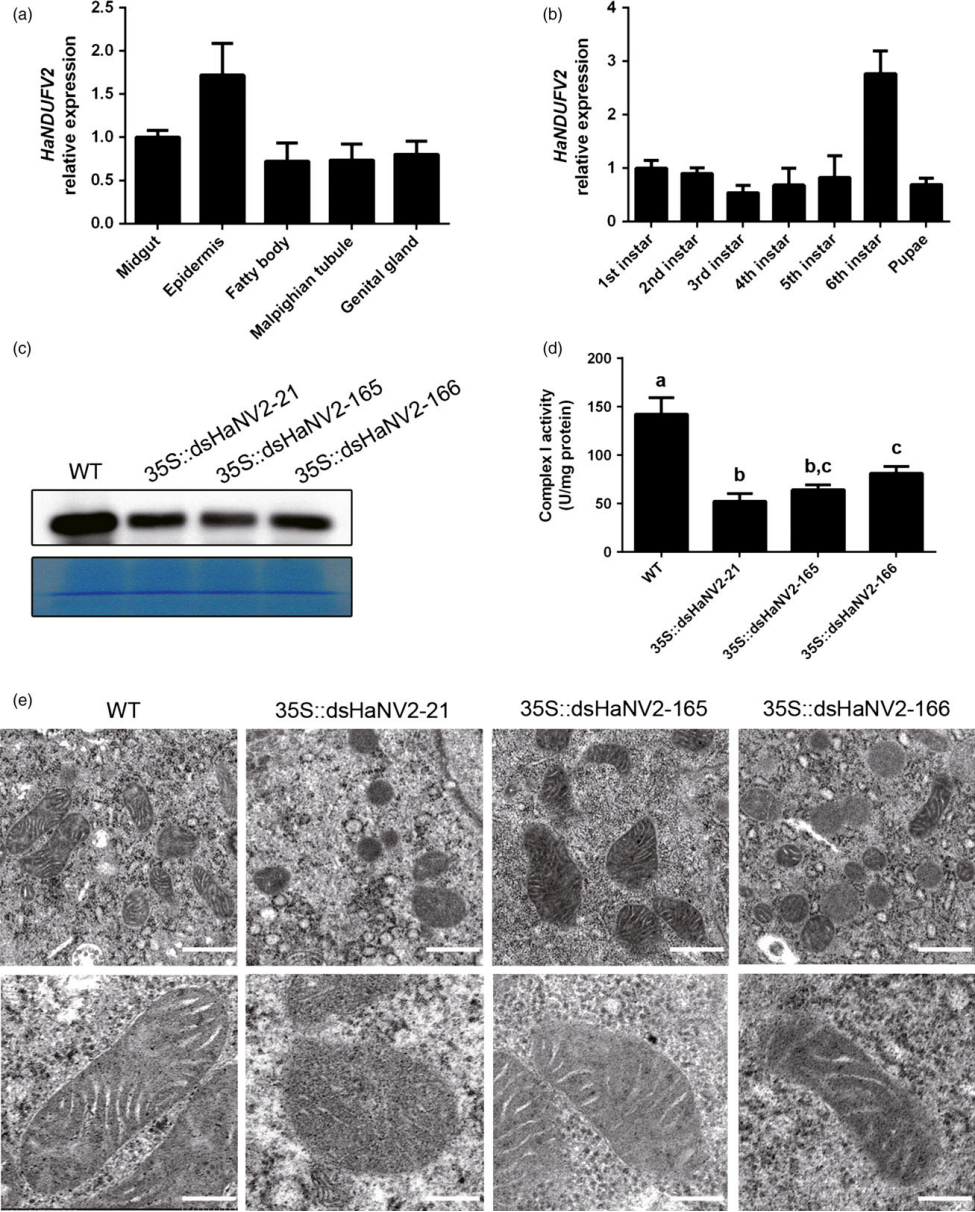
Altered structure and function of *Helicoverpa armigera* mitochondria induced by *HaNDUFV2* suppression. (a) Expression of *HaNDUFV2* in different organs of the 6th‐instar larvae. (b) Expression of *HaNDUFV2* in larvae at different developmental stages. (c) Western blot of HaNDUFV2 protein in midgut of late 2nd‐instar larvae after feeding WT or *35S::dsHaNV2* cotton leaves for 2 days. (d) Complex I activities in midgut of the 2nd‐instar larvae after feeding WT or *35S::dsHaNV2* cotton leaves for 3 days. Data are mean ± SD of three biological replicates. Shared lowercase letters indicate no significant difference between the groups by one‐way ANOVA with Tukey's HSD test at confidence level of *P* < 0.05. (e) Transmission electron micrographs of intestinal wall hair cells in midgut of the larvae after feeding the indicated cotton leaves for 3 days. Scale bars are 500 nm (upper row) or 200 nm (lower row).

As a housekeeping gene, *NDUFV2* was expressed throughout the bollworm larva including midgut, epidermis, malpighian tubules, fatty body and ovary, with the highest transcript abundance in epidermis (Figure 3a), and the expression level did not change significantly throughout the development stages examined, except for a particularly high level at 6th instar (Figure 3b).

To gain insight into the mechanism of insect death triggered by *HaNDUFV2* suppression, we analysed the larvae after 2 days of feeding assay when the growth inhibition started to be evident. Western blot showed that the HaNDUFV2 protein level was reduced in midgut of the larvae that ingested *35S::dsHaNV2* cotton leaves (Figure 3c), which led to decreased complex I activity (Figure 3d). Furthermore, transmission electron microscopic examination of intestinal wall hair cells revealed abnormal morphological structures after *HaNDUFV2* suppression: while the mitochondria in larvae of the control group were elongated and regularly spaced, and the cristae were flattened, those of the *HaNDUFV2*‐suppressed larvae were round with swollen and irregular cristae (Figure 3e). These data highlight the importance of maintaining a critical level of HaNDUFV2 in mitochondrial development and function, and the fatal consequence of *NDUFV2* repression.

**Figure 4:**
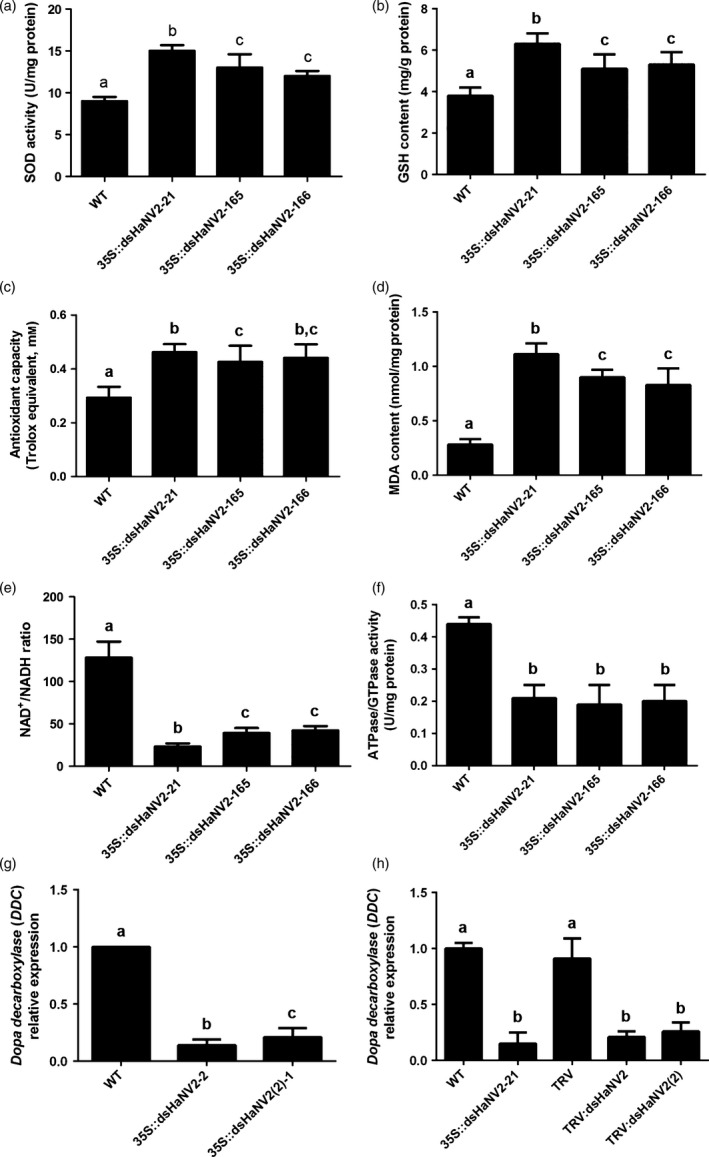
Suppression of *HaNDUFV2* led to altered redox status in *Helicoverpa armigera* midgut. (a) SOD activities in midgut of the 2nd‐instar larvae after feeding WT or *35S::dsHaNV2* cotton leaves for 3 days. Data are mean ± SD of three biological replicates. (b) Increased glutathione (GSH) content in midgut of *H. armigera* larvae as shown in (a). Data are mean ± SD of three biological replicates. (c) Antioxidant capacity (Trolox equivalent) in midgut of *H. armigera* larvae as shown in (a). Data are mean ± SD of three biological replicates. (d) MDA contents in midgut of the larvae as shown in (a). Data are mean ± SD of three biological replicates. (e) NAD ^+^/NADH ratio in midgut of *H. armigera* larvae as shown in (a). Data are mean ± SD of three biological replicates. (f) ATPase activities in midgut of the larvae as shown in (a). Data are mean ± SD of three biological replicates. (g) Expression of *dopa decarboxylase* (*DDC*) gene in *H. armigera* larvae fed with leaves of the wild‐type (WT), *35S::dsHaNV2‐2* or *35S::dsHaNV2(2)‐1 Arabidopsis*. Expression of *DDC* was analysed by qRT‐PCR after 5 days of feeding. Data are mean ± SD of three biological replicates. (h) Expression of *dopa decarboxylase* (*DDC*) gene in midgut of 2nd‐instar larvae after feeding leaves from WT,* 35S::dsHaNV2‐21* and virus‐infected [*TRV:dsHaNV2* and *TRV:dsHaNV2(2)*] cottons for 2 days. Data are mean ± SD of three biological replicates. Shared lowercase letters on panels (a–h) indicate no significant difference between the groups by one‐way ANOVA with Tukey's HSD test at confidence level of *P* < 0.05.

Mitochondria is a major source of reactive oxygen species (ROSs) (Hirst, 2013). The NDUFV2 subunit is involved in controlling ROS generation, and loss of NDUFV2 function led to increased ROSs (Sazanov and Hinchliffe, 2006; Sing *et al*., 2014). We found that suppression of *HaNDUFV2* expression led to increased superoxide dismutase (SOD) activities (Figure 4a) and glutathione (reduced, GSH) contents (Figure 4b), which are important antioxidants in animal cells. Although this led to the increased amount of total antioxidant molecules (Figure 4c), the content of malondialdehyde (MDA), a resultant product of lipid peroxidation and degradation by oxidative damage, still increased (Figure 4d), indicating severe oxidative damage in cells. Moreover, the NAD^+^/NADH ratio, which reflects the balance between oxidized and reduced forms of nicotinamide adenine dinucleotide, decreased significantly in midgut of those fed on transgenic plants (Figure 4e). As NAD^+^ and NADH play an important role in transcriptional regulation, longevity and age‐associated diseases, and the NAD^+^/NADH ratio indicates redox status and metabolism activity in cells and is an important controlling factor of the activity of several key enzymes, including glyceraldehyde 3‐phosphate dehydrogenase and pyruvate dehydrogenase (Lin and Guarente, 2003; Sun *et al*., 2012), decrease in this ratio should cause an changed status of cell metabolism and energy production. Furthermore, the activities of total ATPases/GTPases, which play a key role in energy production and signal transduction, were decreased (Figure 4e).

To gain a global view of the gene expression changes caused by *HaNDUFV2* suppression, midgut transcriptomes of the bollworms fed with WT or *35S::dsHaNV2* cotton leaves for 2 days were generated by RNA‐seq and compared. Assembly of all reads resulted in 32 019 unigenes, of which 26 700 could be functionally annotated. Totally, 11 037 differentially expressed genes (|log_2_Ratio| > 1) were detected, of which 3066 were up‐ and 7971 down‐regulated in the transgenic cotton treated larvae (Table S2). Although this short period feeding reduced the *HaNDUFV2* transcript by only about 36%, altered expressions of genes related to respiratory chain, energy production and metabolite transportation were observed (Table S3), which is consistent with the disruption of mitochondrial structure and function. Interestingly, genes encoding dopa decarboxylase (DDC), which catalyses the production of neural transmitters of dopamine and serotonin and plays a role in insect behaviour, parasite defence and cuticle hardening (Hodgetts and O'Keefe, 2006), were down‐regulated significantly in the larvae treated with *dsHaNV2*‐expressing *Arabidopsis* and cotton (Figure 4f,g, Table S4), implying less dopamine production and decreased neural activities. These results suggest that suppressed *NDUFV2* expression and reduced complex I activities after ingestion of *35S::dsHaNV2* plants led to mitochondria dysfunctions, causing insufficient energy supplement, increased oxidative stress in cells and possibly reduced neural activities due to decreased dopamine production, which, finally, resulted in larvae death.

### Lethal effect by *NDUFV2* suppression is independent of phytoalexins in cotton

Cotton plants accumulate a high concentration of cadinene‐type sesquiterpenoids (e.g. gossypol and hemigossypolone) in epidermal pigmented glands of aerial organs and epidermal/subepidermal layers of root, which function as phytoalexins against herbivores and pathogens (Luo *et al*., 2001; Ma *et al*., 2016; Tan *et al*., 2000; Yang *et al*., 2013). Previously, we used the bollworm P450 gene *CYP6AE14* to develop PM‐RNAi (Mao *et al*., 2007). As it relies on the presence of gossypol in diet, this is most effective on herbivores living on cotton plants. To find out whether the lethal effect of *NDUFV2* repression was also enhanced by gossypol, we employed tobacco rattle virus (TRV) to produce dsRNAs in glanded (gossypol‐containing) and glandless (gossypol‐free) cotton leaves by integrating the target dsRNA fragment into the virus genome, in a manner similar to virus‐induced gene silencing (VIGS) (Kumar *et al*., 2012). Feeding of *H. armigera* larvae with *TRV:dsHaNV2*‐infected glanded cotton leaves for 5 days resulted in a similar adverse effect on larvae survival rate and growth as that observed with stably transformed plants (Figure S4 a,b and c), and a prolonged feeding led to all death of the tested populations, suggesting that this virus‐mediated dsRNA expression in plants mimicked the stable transgenic plants. Importantly, the glanded and the glandless cottons infected with *TRV:dsHaNV2* did not show statistically significant difference on larvae survival and growth rates (Figure S4 a,b), suggesting that insect control by *NDUFV2* suppression is independent of gossypol and possibly other defensive metabolites either, and could be applied to other crops.

### PM‐RNAi of *NDUFV2* is sequence specific and effective to other insect pests

**Figure 5:**
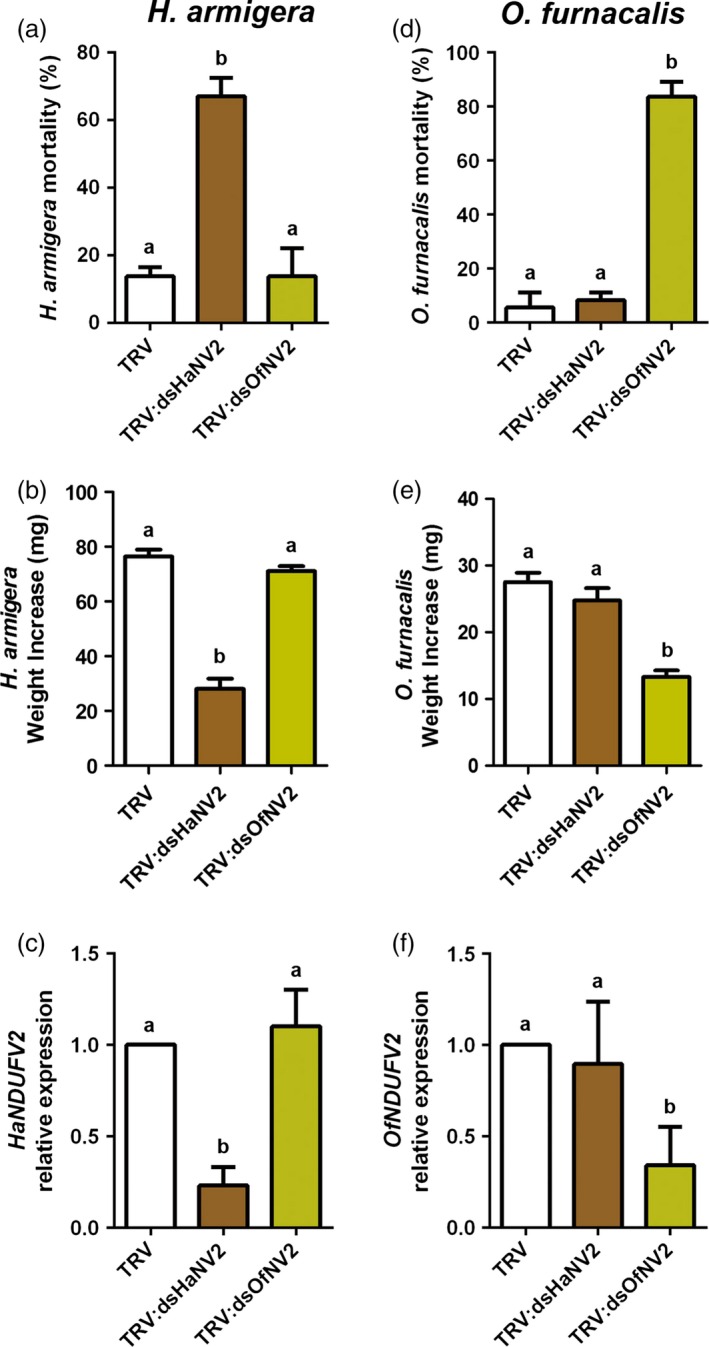
PM‐RNAi of *NDUFV2* was sequence specific. (a) *Helicoverpa armigera* larvae mortality after feeding virus‐inoculated tobacco leaves (*TRV*,*TRV:dsHaNV2* and *TRV:dsOfNV2*) for 5 days. Each treatment started with 18 larvae. Data are mean ± SD of three biological replicates. (b) Weight increase of survived *H. armigera* larvae after feeding virus‐inoculated tobacco leaves (*TRV*,*TRV:dsHaNV2* and *TRV:dsOfNV2*) for 5 days. Each treatment started with 18 larvae. Data are mean ± SD of survivals of three replicates. (c) Expression of *HaNDUFV2* gene in *H. armigera* larvae midgut after feeding virus‐inoculated tobacco leaves for 5 days. Data are mean ± SD of three biological replicates. (d) *Ostrinia furnacalis* larvae mortality after feeding virus‐inoculated tobacco leaves (*TRV, TRV:dsHaNV2* and *TRV:dsOfNV2*) for 5 days. Each treatment started with 18 larvae. Data are mean ± SD of three biological replicates. (e) Weight increase of survived *O. furnacalis* larvae after feeding virus‐inoculated tobacco leaves (*TRV*,*TRV:dsHaNV2* and *TRV:dsOfNV2*) for 5 days. Each treatment started with 18 larvae. Data are mean ± SD of survivals of three replicates. (f) Expression of *OfNDUFV2* in *O. furnacalis* larvae midgut after feeding virus‐inoculated tobacco leaves for 5 days. Data are mean ± SD of three biological replicates. Shared lowercase letters on panels (a–f) indicate no significant difference between the groups by one‐way ANOVA with Tukey's HSD test at confidence level of *P* < 0.05.

We next tested the specificity of *NDUFV2* suppression by PM‐RNAi, which is of particular importance as complex I is a conserved component in respiratory chain of eukaryotes (Brandt, 2006; Hirst, 2013). The Asian corn borer (*Ostrinia furnacalis*) is another lepidopteran insect, and its *NDUFV2* (*OfNDUFV2*) shares nucleotide sequence identity of 79% with *HaNDUFV2* (Figure S5). A homologous fragment from *OfNDUFV2*, which shares nucleotide identity of 80% with that used for *HaNDUFV2* RNAi (Figure S5), was used to generate the *TRV:dsOfNV2* construct. Tobacco (*Nicotiana benthamiana*) leaves expressing either dsRNAs were used to feed cotton bollworms or Asian corn borers for 5 days. We found that, after ingesting leaves expressing their own *dsNDUFV2*, both *H. armigera* and *O. furnacalis* showed high mortality of about 70% (Figure 5a,d) and significantly reduced weight of the survivals (Figure 5b,e). Consistently, *NDUFV2* expression levels in midgut of each species were decreased (Figure 5c,f). However, when exchanged, that is feeding *H. armigera* with *TRV:dsOfNV2* leaves and *O. furnacalis* with *TRV:dsHaNV2* leaves, neither insect showed a significant difference in mortality and weight increase from the control group, nor the change in expression levels of the respective *NDUFV2* gene (Figure 5a–f). These results indicate that *NDUFV2* can be extended to other lepidopteran chewing insects, and, importantly, without observable cross‐activities, at least at the level of 80% nucleotide sequence identity.

**Figure 6:**
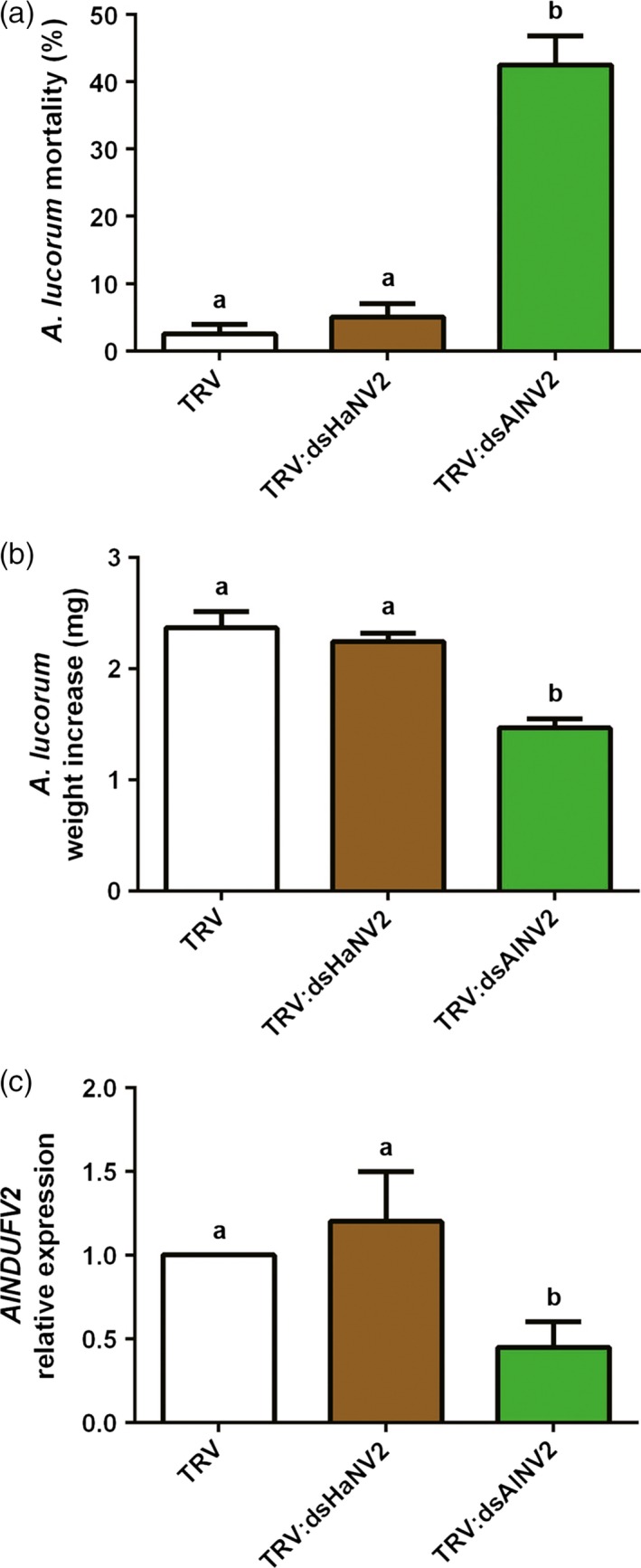
PM‐RNAi of *NDUFV2* was effective against *Apolygus lucorum*. (a) Mortality of *A. lucorum* nymphs after feeding virus‐inoculated cotton leaves (*TRV*,*TRV:dsHaNV2* and *TRV:dsAlNV2*) for 7 days. Each treatment started with 20 nymphs. Data are mean ± SD of three biological replicates. (b) Weight increase of *A. lucorum* nymphs after feeding of *TRV*,*TRV:dsHaNV2* and *TRV:dsAlNV2* leaves for 7 days. Each treatment started with 20 larvae. Data are mean ± SD of survivals of three replicates. (c) *AlNDUFV2* gene expression after feeding of *TRV*,*TRV:dsHaNV2* and *TRV:dsAlNV2* leaves for 7 days. Total RNAs were extracted from pooled nymphs, and *AlNDUFV2* gene expression was analysed by qRT‐PCR. Data are mean ± SD of three biological replicates. Shared lowercase letters on panels (a), (b) and (c) indicate no significant difference between the groups by one‐way ANOVA with Tukey's HSD test at confidence level of *P* < 0.05.

Lygus (*Apolygus lucorum*), a species of the Miridae family, has become an increasingly devastating insect pest in field in recent years, correlating to the wide cultivation of Bt crops and thus the reduced insecticide usage (Lu *et al*., 2010, 2012). They puncture plant tissues with piercing‐sucking mouthparts and feed by sucking sap, in contrast to chewing insects of cotton bollworm and corn borer. Placing *A. lucorum* on *TRV:AlNV2*‐infected cotton plants for 7 days also resulted in the decrease in nymph numbers (or increased mortality) and retarded growth, compared with the group on the control (empty vector) plants (Figure 6a–c). And similarly, no growth inhibitory effect was detected in the group fed with *TRV:dsHaNV2* cotton plants. These results suggest that PM‐RNAi against *NDUFV2* is also effective in controlling lygus.

### Genes of other subunits of complex I can also be effective in PM‐RNAi

**Figure 7:**
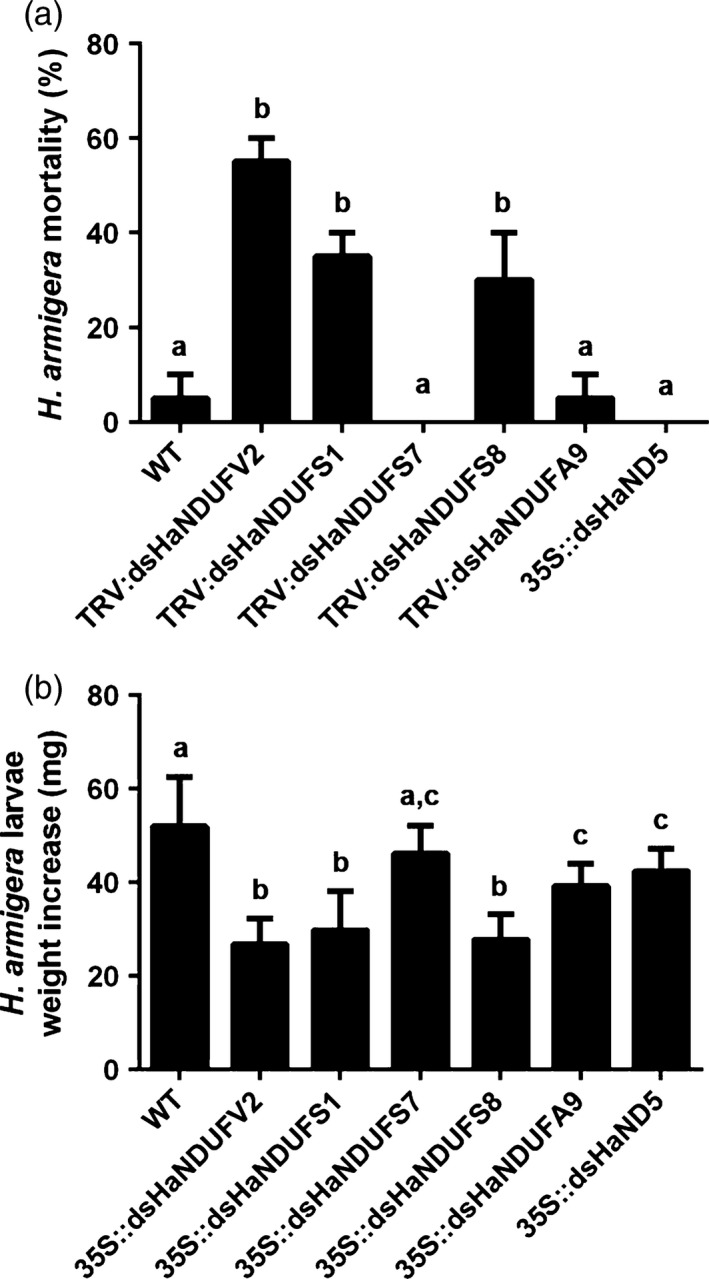
PM‐RNAi of other subunit genes of mitochondrial complex I of *Helicoverpa armigera*. (a) Mortality of larvae after feeding TRV‐infected cotton leaves expressing dsRNAs targeting five mitochondrial complex I subunits, respectively, for 5 days. Each treatment started with 18 larvae. Data are mean ± SD of three biological replicates. (b) Weight increase of survived larvae after a 5‐day feeding on TRV‐infected cotton leaves expressing dsRNAs targeting different mitochondrial complex I subunits. Each treatment started with 18 larvae. Data are mean ± SD of survivals of three replicates. Shared lowercase letters on panels (a) and (b) indicate no significant difference between the groups by one‐way ANOVA with Tukey's HSD test at confidence level of *P* < 0.05.

To find out whether other subunits of complex I could be used as PM‐RNAi targets, we searched *H. armigera* EST data set and identified the nuclear genes encoding subunits of NDUFV1, NDUFS1, NDUFS2, NDUFS3, NDUFS7, NDUFS8 and NDUFAF1, and the mitochondrial genes encoding subunits of ND1, ND4 and ND5. Feeding assays with TRV‐mediated dsRNA expressing cotton plants and transgenic *Arabidopsis* plants revealed that, although HaNDUFV2 appeared to be the most potent, *HaNDUFS1* and *HaNDUFS8* were also highly active in inducing mortality and larval growth retardation (Figure 7a,b).

Dysfunctions of mitochondrial complex I subunits have been reported to be correlated to the imbalance of NADH/NAD+ ratio and ROS production (Kussmaul and Hirst, 2006), causing lactic acidosis and neurological disorders like mitochondrial encephalomyopathy (Benit *et al*., 2003; Koene *et al*., 2012). We found that the suppression of *NDUFV2* by PM‐RNAi, which led to the disruption of mitochondrial structures and cellular functions in insect midgut, can be used as a highly effective and specific approach for insect pest control. We demonstrate that more than lepidopterans which can be controlled by Bt toxins, the Bt‐insensitive insect pests, such as the lygus of hemipteran, are also susceptible to plant‐mediated *NDUFV2* RNAi. Targeting insect mitochondrial complex I is expected to promote the development of the field application of RNAi as next‐generation insect‐proof crops.

## Material and methods

### Culture of plants and insects

Cotton (*Gossypium hirsutum* cv. R15, *35S::dsHaNV2* transgenic and virus inoculated) plants were grown in a climate chamber at 28 °C with 60–80% relative humidity. *Arabidopsis thaliana* (ecotype Col‐0 and transgenic) and tobacco (*Nicotiana benthamiana*) plants were grown at 22 °C, all on a 16‐h day/8‐h night photoperiod. Cotton bollworm (*H. armigera*) and Asian corn borer (*O. furnacalis*) were reared in the laboratory at 25 °C and 70% relative humidity with 14‐h photoperiod on a modified artificial diet (Wu and Gong, 1997), and lygus (*A. lucorum*) on kidney beans.

### Vector construction and plant transformation

For constitutive expression of dsRNAs in *Arabidopsis* and cotton, the fragment of *HaNDUFV2* gene (197–560 bp in ORF) was amplified and inserted to the binary vector pCAMBIA2301 in sense and antisense direction interspaced by a 120‐bp intron of *A. thaliana RTM1* gene (Mao *et al*., 2007) to produce the construct *35S::dsHaNV2*, or into vector pYL156 to produce the virus expressing dsRNA construct *TRV:dsHaNV2*. The region of 501–755 bp was used to produce the *35S::dsHaNV2(2)* construct. Primers for constructs are listed in Table S5. The constructs were transferred to *Agrobacterium tumefaciens* strains GV3101 (for *Arabidopsis*) and LBA4404 (for cotton) by electroporation. *A. thaliana* (Col‐0) was transformed using the floral dip method (Clough and Bent, 1998), and transgenic cotton was generated as described (Shangguan *et al*., 2008).

### Virus‐mediated dsRNA expression

*Agrobacterium* harbouring pTRV1 or the modified pYL156 containing dsRNA fragments were grown at 28 °C in a shaking incubator overnight. Cells were collected and suspended in 10 mm MES, 10 mm MgCl_2_ and 200 μm acetylsyringone solution to a final OD600 = 2.0 and then left at room temperature for 4 h. Mixed *A. tumefaciens* suspensions of pTRV1 and an equal volume of pYL156 or its derivatives were infiltrated into cotyledons of 10‐day‐old cotton seedlings, or 1‐month‐old tobacco leaves. Thirty days after inoculation, fully expanded true leaves were collected for analysis or insect feeding assay.

### Insect feeding assay

New and fully expended leaves from 1‐month‐old *Arabidopsis* or 4‐month‐old cotton were used for feeding assays. For each experiment, the synchronous 2nd‐instar larvae were selected and divided into three groups, each contained 18 individuals, unless otherwise indicated. After feeding on diets for indicated days, larvae were weighed and midgut was collect for further analysis. For lygus feeding assay, the 2nd‐instar nymphs (20 per treatment) were reared with five cotton plants in a container for 7 days. After assay, nymphs were collected to determine survival rate and body weight. Statistical data analysis was performed using one‐way ANOVA following Tukey's HSD (honest significant difference) test with SPSS software IBM, Armonk, New York, USA.

### RNA extraction and analysis

Total RNAs from cotton or *Arabidopsis* plants were extracted with a CTAB method (Zhao *et al*., 2012). Insect RNAs were extracted with TRIzol reagent (Ambion; Carlsbad, CA) according to the manufacturer's instructions. One microgram of DNase I‐treated total RNAs was reverse‐transcribed using the SuperScript III First‐Strand Synthesis SuperMix and oligo‐dT or random primers (Life Technologies, Waltham, MA, USA). qRT‐PCR was performed with SYBR‐Green PCR Mastermix (TaKaRa, Dalian, China) on a cycler (Mastercycler RealPlex; Eppendorf, Hamburg, Germany) with primers listed in Table S5. Each biological replicate contained at least three technical replicates of the qPCR assay.

For analysis of small RNAs, the total RNA isolated from cotton leaves was precipitated with 50% isopropanol and then solubilized in water. High molecular weight RNA was precipitated with 30% polyethylene glycol (PEG) 8000 (Sigma, St. Louis, MO, USA), followed by precipitation of low molecular weight RNA by twice volumes of ethanol. The low molecular weight RNA was separated by 17% denaturing polyacrylamide gel and transferred to Hybond N^+^ nylon membrane (GE Healthcare, Little Chalfont, UK). The membrane was probed with biotin labelled U6 or *HaNDUFV2* nucleotide and detected with Chemiluminescent Nucleic Acid Detection Module Kit (89880; Thermo Scientific, Waltham, MA, USA) according to the manufacturer's instruction.

### Transcriptome analysis

Total RNAs from midgut of cotton bollworm larvae fed on WT or *35S::dsHaNV2* cotton leaves for 2 days were extracted, and mRNAs were separated using oligo‐dT magnetic beads and sheared into short fragments (E200 bp) in the fragmentation buffer. The double‐stranded cDNAs were synthesized and purified with a QiaQuick PCR extraction kit (Qiagen, Hilden, Germany). After end repair and ligation of sequencing adaptors, the fragments were purified and sequenced with a high‐throughput sequencer (HiSeq 2000; Illumina, San Diego, CA, USA) with paired‐end read length of 200 bp. Three biological replicates were performed separately. After filtration, cleaned reads were assembled using SOAPdenovo (BGI, Beijing, China). Gene expression levels were normalized and calculated as reads per kb per million reads values.

### Western blot analysis

After the feeding assay, larval midgut tissues were collected from each group, grinded in liquid nitrogen and extracted with protein extraction buffer (50 mm Tris, pH 7.4; 1% SDS) (Sangon Biotech, Shanghai, China) and centrifuged for 5 min at 4 °C to harvest the supernatant. Protein concentration was determined using the Thermo Scientific protein assay kit. Twenty micrograms of the extracted proteins was loaded per lane onto 10% Tris‐glycine gels for electrophoresis and electro‐transferred to nitrocellulose membrane (Life Technologies). The membrane was blocked with 5% nonfat dry milk in PBST buffer (C520004, Sangon Biotech, Shanghai, China) for 1 h at room temperature, followed by incubation with anti‐NDUFV2 antibody (HPA003404, Sigma, St. Louis, MO, USA) for 16 h at 4 °C. After washing with PBST buffer, the membrane was incubated with horseradish peroxidase (HRP)‐conjugated anti‐rabbit antiserum (G‐21234, Life Technology, Waltham, MA, USA) for 1 h at room temperature. The membrane was developed using SuperSignal West Pico Chemiluminescent Substrate (Life Technologies, Waltham, MA, USA).

### Transmission electron microscopy

The 2nd‐instar larvae of *H. armigera* were reared on the transgenic or nontransgenic cotton leaves for 3 days, and midgut was detached by a sharp double‐edge blade and immediately fixed in formalin–acetic acid–alcohol (FAA) solution. TEM was performed with HITACHI H‐7650.

### Enzyme activity assays

Midgut was taken from each larva and washed with an ice‐cold 0.9% NaCl solution. Mitochondria isolation from larval midgut and complex I activity assay were performed as described (Frezza *et al*., 2007; Janssen *et al*., 2007). The ATPase and superoxide dismutase (SOD) activities were detected with ATPase/GTPase Activity Assay Kit (Sigma) and SOD Assay Kit (Sigma), respectively. The MDA and GSH contents were determined with Lipid Peroxidation (MDA) Assay Kit (Sigma) and Glutathione Assay Kit (Sigma). Total antioxidant capacity assay was performed with Antioxidant Assay Kit (CS0790, Sigma), and NAD^+^/NADH ratio was determined by the NAD/NADH Quantification Kit (MAK037, Sigma).

## Competing financial interests

The authors declare no competing financial interests.

## Supporting information


**Figure S1** Inhibition of cotton bollworm (*Helicoverpa armigera*) larval growth by *35S::dsHaNV2 Arabidopsis* leaves.
**Figure S2** Northern blot analysis of *NDUFV2* small RNAs in *35S::dsHaNV2* transgenic cotton plants.
**Figure S3** Decreased growth and survival rates of cotton bollworm larvae after feeding *35S::dsHaNV2* cotton leaves.
**Figure S4** Lethal effect of *NDUFV2* suppression was independent of phytoalexins in cotton.
**Figure S5** Alignment of nucleotide sequences of *NDUFV2* ORFs from *Helicoverpa armigera* (*HaNDUFV2*), *Ostrinia furnacalis* (*OfNDUFV2*), *Apolygus lucorum* (*AlNDUFV2*) and human (*HsNDUFV2*).

**Table S1** Pupation and Eclosion status of *Helicoverpa armigera* fed with WT or *35S::dsHaNV2* transgenic *Arabidopsis* leaves for 30 days.
**Table S4** FPKM values of genes encoding dopa decarboxylase in cotton bollworm larvae (late 2nd‐instar) after feeding WT or *35S::dsHaNDUFV2* cotton leaves for 2 days.
**Table S5** Oligonucleotide primers used in this investigation.

**Table S2** Pairwise comparison of differentially expressed genes in midgut of *Helicoverpa armigera* fed with WT or *35S::dsHaNV2* cotton leaves.

**Table S3** List of downregulated genes that encode mitochondria localized proteins in *Helicoverpa armigera* fed with *35S::dsHaNV2* cotton leaves.
