# Supplementary material for: Targeting insect mitochondrial complex I for plant protection
Source: Plant Biotechnol J. 2016 Mar 17;14(9):1925–35. doi: 10.1111/pbi.12553 (PMC5069633; doi:10.1111/pbi.12553)
Supplement: Supplementary file 1 — Figure S1 Inhibition of cotton bollworm (Helicoverpa armigera) larval growth by 35S::dsHaNV2 Arabidopsis leaves. Figure S2 Northern blot analysis of NDUFV2 small RNAs in 35S::dsHaNV2 transgenic cotton plants. Figure S3 Decreased growth and survival rates of cotton bollworm larvae after feeding 35S::dsHaNV2 cotton leaves. Figure S4 Lethal effect of NDUFV2 suppression was independent of phytoalexins in cotton. Figure S5 Alignment of nucleotide sequences of NDUFV2 ORFs from Helicoverpa armigera (HaNDUFV2), Ostrinia furnacalis (OfNDUFV2), Apolygus lucorum (AlNDUFV2) and human (HsNDUFV2). [file PBI-14-1925-s003.docx]

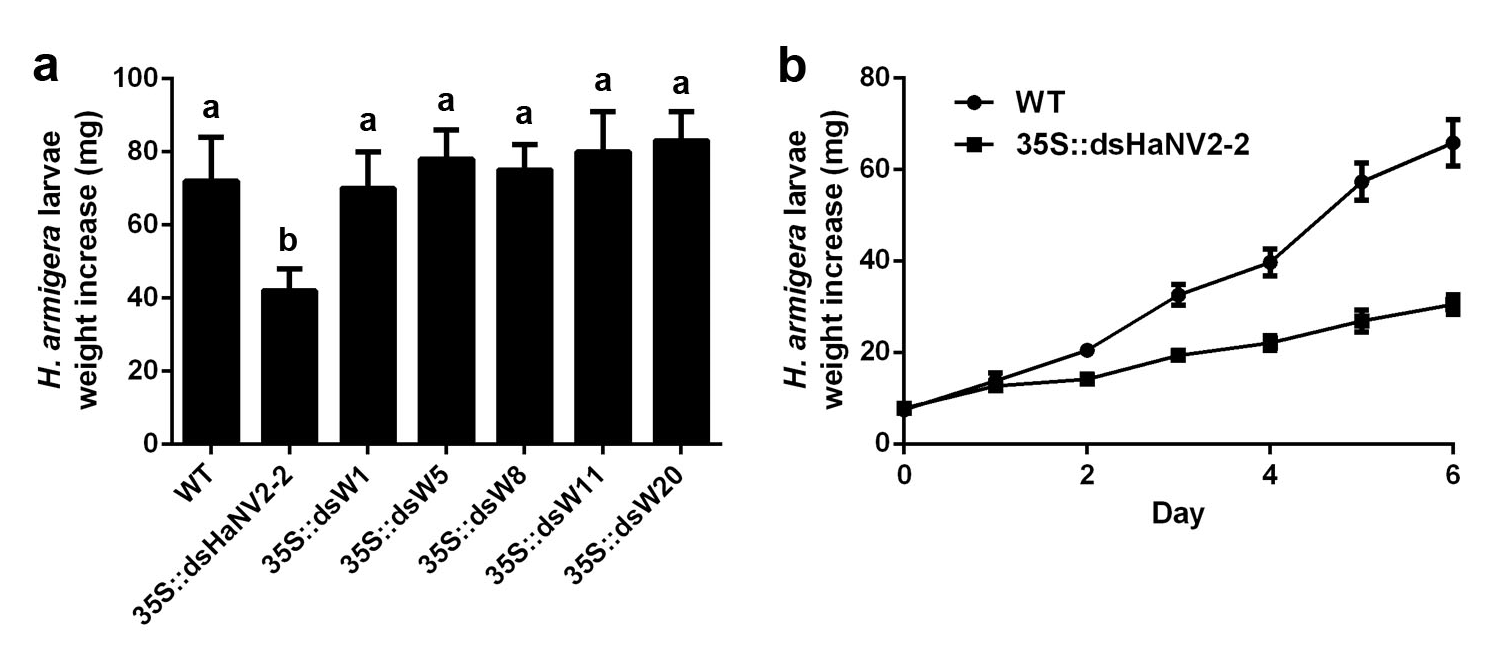


**Figure S1.** Inhibition of cotton bollworm (*H. armigera*) larval growth by *35S::dsHaNV2* *Arabidopsis* leaves. (a) Weight increase of larvae after feeding *Arabidopsis* leaves expressing dsRNAs targeting to bollworm genes for 5 days. *35S::dsW1*-*35S::dsW20* represent *Arabidopsis* plants engineered to express the dsRNA corresponding to *W1*-*W20* genes, under the control of 35S promoter. W1: chymotrypsin; W5: juvenile hormone diol kinase; W8: ATP synthase beta subunit; W11: actin; W20: mitochondrial ATP synthase alpha subunit precursor. Each treatment started with 18 larvae. Data are mean ± SD of survivals of three replicates. Shared lowercase letters indicate no significant difference between the groups by one-way ANOVA with Tukey HDS test at confidence level of P < 0.05. (b) Time course of larval weight fed with WT or *35S::dsHaNV2* *Arabidopsis* leaves.


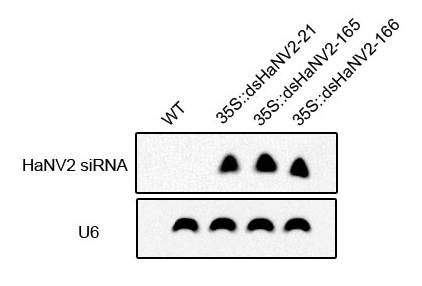


**Figure S2.** Northern blot analysis of *NDUFV2* small RNAs in *35S::dsHaNV2* transgenic cotton plants.

Low-molecular-weight RNAs were extracted from WT and *35S::dsHaNV2* transgenic cotton leaves. About 50 μg RNA was separated by 17% denaturing polyacrylamide gel and transferred to Hybond N^+^ nylon membrane, and probed with biotin labeled U6 or *HaNDUFV2* nucleotide.


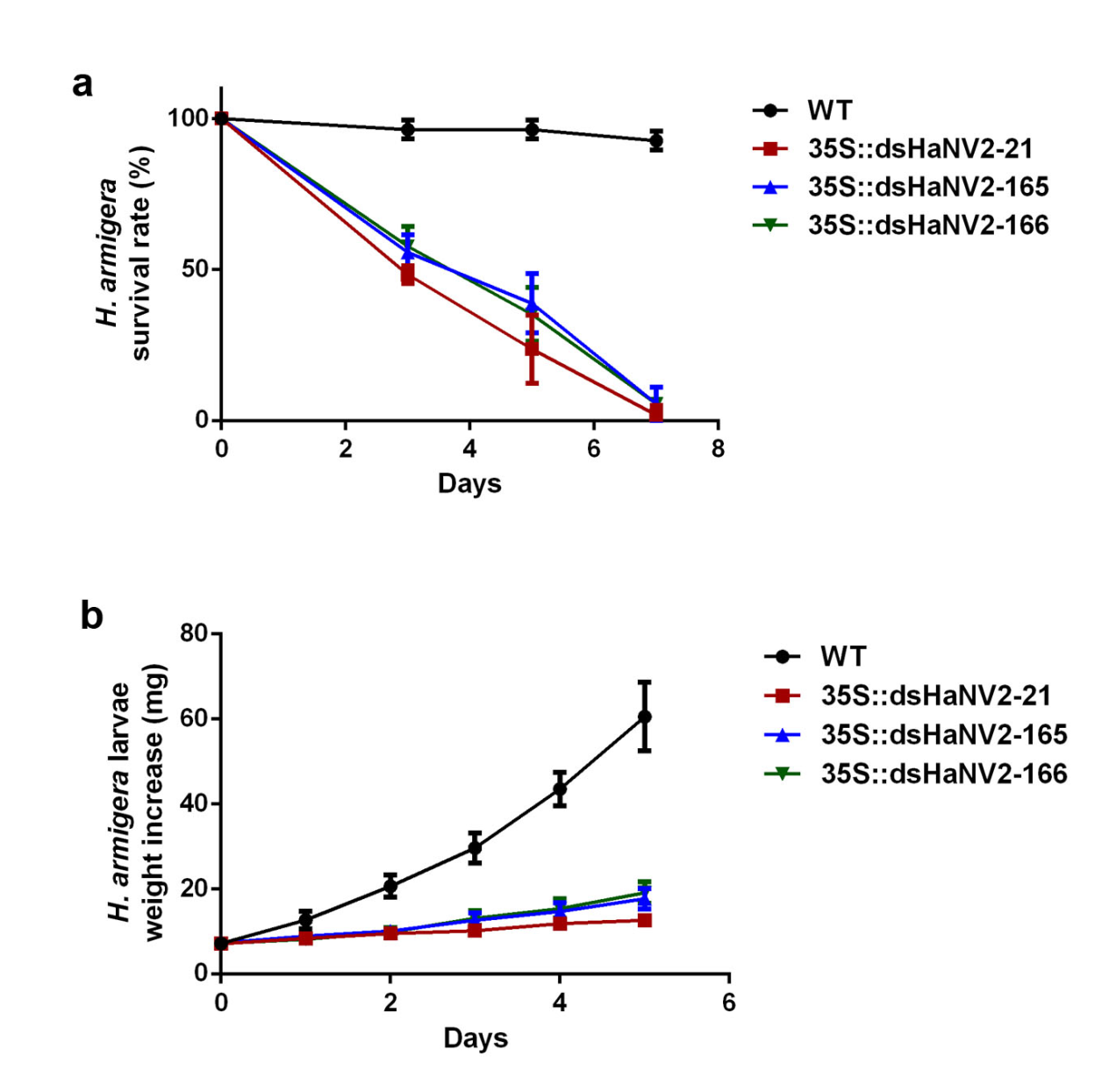


**Figure S3.** Decreased growth and survival rates of cotton bollworm larvae after feeding *35S::dsHaNV2* cotton leaves.

Three *35S::dsHaNV2* lines of cotton (*G. hirsutum*) and the non-transgenic cotton (WT) were used. Almost all larvae on *35S::dsHaNV2* leaves died at day 7 (a) and exhibited little growth (b). Each group started with 18 larvae, and the weight of the survived larva was recorded. Data are mean ± SD of three biological replicates.


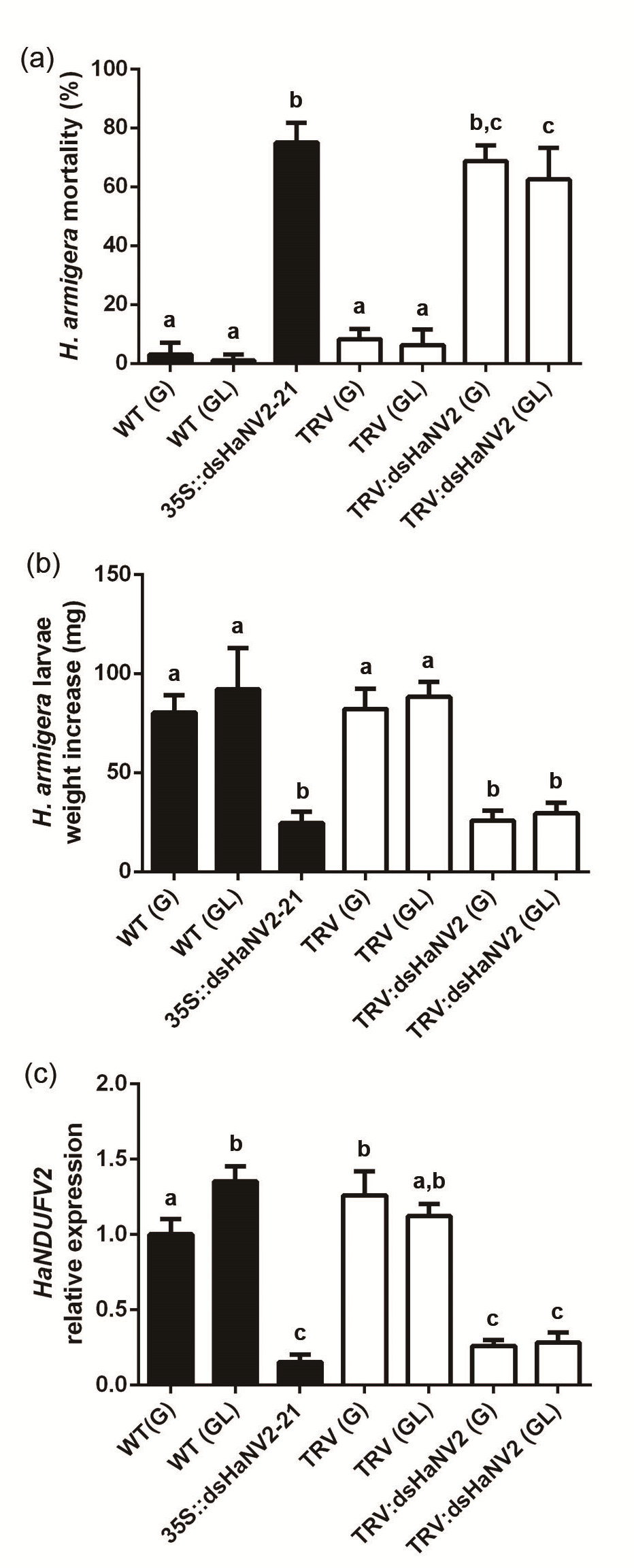


**Figure S4.** Lethal effect of *NDUFV2* suppression was independent of phytoalexins in cotton. (a) Mortality of *H. armigera* larvae fed with leaves from non-transgenic, stably transformed or TRV infected cotton leaves for 5 days. Both glanded (G) and glandless (GL) cottons were inoculated with virus (TRV or TRV:dsHaNV2) before feeding the 2nd-instar larvae. The *35S::dsHaNV2-21* transgenic cotton is glanded (G). Each treatment started with 18 larvae. Data are mean ± SD of three biological replicates. (b) Weight increase of *H. armigera* larvae after feeding leaves of glanded (G) and glandless (GL) cotton cultivars, or those inoculated with virus (TRV or TRV:dsHaNV2). Each treatment started with 18 larvae. Data are mean ± SD of survivals of three replicates. (c) Expression of *HaNDUFV2* in larvae after feeding glanded and glandless cottons as well as those inoculated with the virus. Data are mean ± SD of three biological replicates.

Shared lowercase letters on panels (a), (b) and (c) indicate no significant difference between the groups by one-way ANOVA with Tukey HDS test at confidence level of P < 0.05.


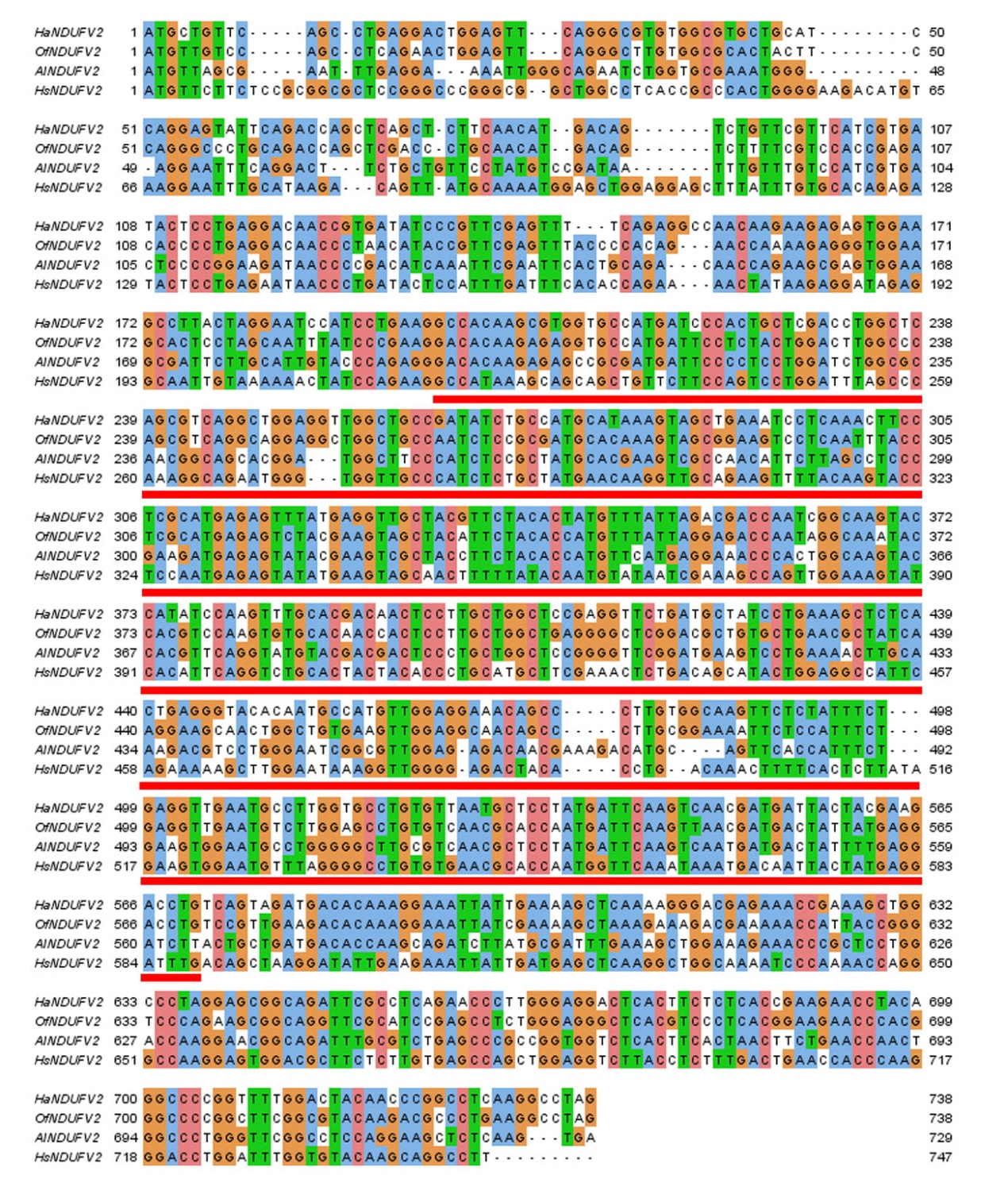


**Figure S5.** Alignment of nucleotide sequences of *NDUFV2* ORFs from *H. armigera* (*HaNDUFV2*), *O. furnacalis* (*OfNDUFV2*), *A. lucorum* (*AlNDUFV2*) and human (*HsNDUFV2*).

Red underline represents the common region used to produce dsRNAs for feeding assays of *H. armigera*, *O. furnacalis* and *A. lucorum*.
