# Supplementary material for: Targeting insect mitochondrial complex I for plant protection
Source: Plant Biotechnol J. 2016 Mar 17;14(9):1925–35. doi: 10.1111/pbi.12553 (PMC5069633; doi:10.1111/pbi.12553)
Supplement: Supplementary file 2 — Table S1 Pupation and Eclosion status of Helicoverpa armigera fed with WT or 35S::dsHaNV2 transgenic Arabidopsis leaves for 30 days. Table S4 FPKM values of genes encoding dopa decarboxylase in cotton bollworm larvae (late 2nd‐instar) after feeding WT or 35S::dsHaNDUFV2 cotton leaves for 2 days. Table S5 Oligonucleotide primers used in this investigation. [file PBI-14-1925-s004.docx]

**Table S1.** Pupation and Eclosion status of *H. armigera* fed with WT or 35S::dsHaNV2 transgenic *Arabidopsis* leaves for 30 days.

In each test, 18 larvae were fed with WT or *35S::dsHaNV2* transgenic *Arabidopsis* leaves for 30 days. Final numbers of survivals, pupae and eclosion status were recorded.

|  |  | Initial larvae No. | Survivals | Pupae No. | Moth No. |
| --- | --- | --- | --- | --- | --- |
| WT | Test 1 | 18 | 17 | 15 | 12 |
|  | Test 2 | 18 | 18 | 17 | 13 |
|  | Test 3 | 18 | 16 | 16 | 12 |
| 35S::dsHaNV2-2 | Test 1 | 18 | 0 | 0 | 0 |
|  | Test 2 | 18 | 0 | 0 | 0 |
|  | Test 3 | 18 | 0 | 0 | 0 |
| 35S::dsHaNV2-5 | Test 1 | 18 | 0 | 0 | 0 |
|  | Test 2 | 18 | 0 | 0 | 0 |
|  | Test 3 | 18 | 0 | 0 | 0 |
| 35S::dsHaNV2-7 | Test 1 | 18 | 1 | 1 | 0 |
|  | Test 2 | 18 | 0 | 0 | 0 |
|  | Test 3 | 18 | 2 | 1 | 0 |
| 35S::dsHaNV2-8 | Test 1 | 18 | 1 | 0 | 0 |
|  | Test 2 | 18 | 1 | 1 | 0 |
|  | Test 3 | 18 | 3 | 2 | 0 |

**Table S4.** FPKM values of genes encoding dopa decarboxylase in cotton bollworm larvae (late 2nd-instar) after feeding WT or *35S::dsHaNDUFV2* cotton leaves for 2 days.

| Gene ID | Length (bp) | WT | | 35S::dsHaNDUFV2 | | Fold of decrease |
| --- | --- | --- | --- | --- | --- | --- |
|  |  | AVG | STD | AVG | STD |  |
| Unigene2058 | 1074 | 34.0351 | 9.2871 | 4.4131 | 5.7887 | 0.1296 |
| Unigene2477 | 1558 | 2.2377 | 0.8101 | 0.4788 | 0.0730 | 0.2139 |
| Unigene3317 | 1538 | 7.2731 | 3.7135 | 2.1795 | 1.1197 | 0.2996 |
| Unigene4153 | 1614 | 2.2182 | 0.8391 | 0.4853 | 0.0316 | 0.2188 |
| Unigene4428 | 1630 | 3.2594 | 0.7771 | 0.5394 | 0.0520 | 0.1654 |
| Unigene4989 | 1540 | 5.7778 | 4.0258 | 1.7838 | 0.3016 | 0.3087 |
| Unigene5018 | 1614 | 2.1017 | 0.8105 | 0.4499 | 0.0595 | 0.2140 |
| Unigene5167 | 1649 | 2.1829 | 0.7218 | 0.4638 | 0.0784 | 0.2124 |
| Unigene5863 | 1521 | 2.218 | 0.8448 | 0.4775 | 0.0631 | 0.2153 |
| Unigene5327 | 1659 | 12.522 | 9.9103 | 4.1205 | 1.7259 | 0.3290 |
| CL1680 | 957 | 90.2188 | 32.0929 | 27.9212 | 16.7460 | 0.3094 |

**Table S5.** Oligonucleotide primers used in this investigation.

| **Primer** | **Sequence (5’-3’)** | **Purpose** |
| --- | --- | --- |
| HaNV2-F-BamHI | CGGGATCC GCCACAAGCGTGGTGCCATG | dsRNA, TRV |
| HaNV2-R-XbaI | CGTCTAGA CAGGTCTTCGTAGTAATCAT | dsRNA, TRV |
| HaNV2-F-SacI | CGGAGCTC GCCACAAGCGTGGTGCCATG | dsRNA |
| HaNV2-R-NotI | CGGCGGCCGC CAGGTCTTCGTAGTAATCAT | dsRNA |
| HaNV2(2)-F-BamHI | CGGGATCC GGTTGAATGCCTTGGTGCCT | dsRNA, TRV |
| HaNV2(2)-R-XbaI | CGTCTAGA AACGGAAACTTTTTGTTCTAGG | dsRNA, TRV |
| HaNV2(2)-F-SacI | CGGAGCTC GGTTGAATGCCTTGGTGCCT | dsTNA |
| HaNV2(2)-R-NotI | CGGCGGCCGC AACGGAAACTTTTTGTTCTAGG | dsRNA |
| OfNV2-F-BamHI | CGGGATCC GACACAAGAGAGGTGCCATG | TRV |
| OfNV2-R-XbaI | CGTCTAGA CAGGTCCTCATAATAGTCAT | TRV |
| AlNV2-F-BamHI | CGGGATCC GACACAAGAGAGCCGCGATG | TRV |
| AlNV2-R-XbaI | CGTCTAGA AGATCCTCAAAATAGTCATC | TRV |
| HaNDUFS1-F-BamHI | CGGGATCC TTAGCAGACGCAGAATCGC | dsRNA, TRV |
| HaNDUFS1-R-XbaI | CGTCTAGA GCTGACTCCAGGCGTTTT | dsRNA, TRV |
| HaNDUFS1-F-SacI | CGGAGCTC TTAGCAGACGCAGAATCGC | dsRNA |
| HaNDUFS1-R-NotI | CGGCGGCCGC GCTGACTCCAGGCGTTTT | dsRNA |
| HaNDUFS2-F-BamHI | CGGGATCC CAGCCAGAAGAACGGAGTC | dsRNA, TRV |
| HaNDUFS2-R-XbaI | CGTCTAGA GATGCCGGACGAGGAGTA | dsRNA, TRV |
| HaNDUFS2-F-SacI | CGGAGCTC CAGCCAGAAGAACGGAGTC | dsRNA |
| HaNDUFS2-R-NotI | CGGCGGCCGC GATGCCGGACGAGGAGTA | dsRNA |
| HaNDUFS7-F-BamHI | CGGGATCC CGGATTGGCTTGTTGTGC | dsRNA, TRV |
| HaNDUFS7-R-XbaI | CGTCTAGA CATTTATTACTGCAACAGAGGGT | dsRNA, TRV |
| HaNDUFS7-F-SacI | CGGAGCTC CGGATTGGCTTGTTGTGC | dsRNA |
| HaNDUFS7-R-NotI | CGGCGGCCGC CATTTATTACTGCAACAGAGGGT | dsRNA |
| HaNDUFS8-F-BamHI | CGGGATCC GGATTTGCTGTCACCCTG | dsRNA, TRV |
| HaNDUFS8-R-XbaI | CGTCTAGA AACGGTAGAGGTGGTCAGC | dsRNA, TRV |
| HaNDUFS8-F-SacI | CGGAGCTC GGATTTGCTGTCACCCTG | dsRNA |
| HaNDUFS8-R-NotI | CGGCGGCCGC AACGGTAGAGGTGGTCAGC | dsRNA |
| HaNDUFA9-F-BamHI | CGGGATCC GTTGTGGTCGTTGAAGGC | dsRNA, TRV |
| HaNDUFA9-R-XbaI | CGTCTAGA TGCTCCAGGTTGCGTTT | dsRNA, TRV |
| HaNDUFA9-F-SacI | CGGAGCTC GTTGTGGTCGTTGAAGGC | dsRNA |
| HaNDUFA9-R-NotI | CGGCGGCCGC TGCTCCAGGTTGCGTTT | dsRNA |
| HaND5-F-BamHI | CGGGATCC ATATTTGTATTTTTGATTTCTTCTGT | dsRNA, TRV |
| HaND5-R-XbaI | CGTCTAGA ACCAATACGATTAGATAAAGCAG | dsRNA, TRV |
| HaND5-F-SacI | CGGAGCTC ATATTTGTATTTTTGATTTCTTCTGT | dsRNA |
| HaND5-R-NotI | CGGCGGCCGC ACCAATACGATTAGATAAAGCAG | dsRNA |
|  |  |  |
| HaNV2-F | CAACAAGAAGAGAGTGGAAGC | qRT-PCR |
| HaNV2-R | TTTCGGTTTCTCGTCCCT | qRT-PCR |
| HaDDC-F | CGTCACTGGCAAATACCTCT | qRT-PCR |
| HaDDC-R | CATCAGCAGCAGCCTTTAC | qRT-PCR |
| HaActin-F | AAGTTGCTGCGCTGGTAGTAG | qRT-PCR |
| HaActin-R | AGTTCGTAGGACTTCTCCAGG | qRT-PCR |
| OfNV2-F | GACACAAGAGAGGTGCCATG | qRT-PCR |
| OfNV2-R | CAGGTCCTCATAATAGTCAT | qRT-PCR |
| OfActin-F | CCTTCGTAGATAGGGACGGT | qRT-PCR |
| OfActin-R | GACAACGGCTCCGGTATGT | qRT-PCR |
| AlNV2-F | GACACAAGAGAGCCGCGATG | qRT-PCR |
| AlNV2-R | AGATCCTCAAAATAGTCATC | qRT-PCR |
| AlActin-F | GACTGTCTCTCCGTCCAACT | qRT-PCR |
| AlActin-R | GAATTTGCCAGCACTTTACA | qRT-PCR |
